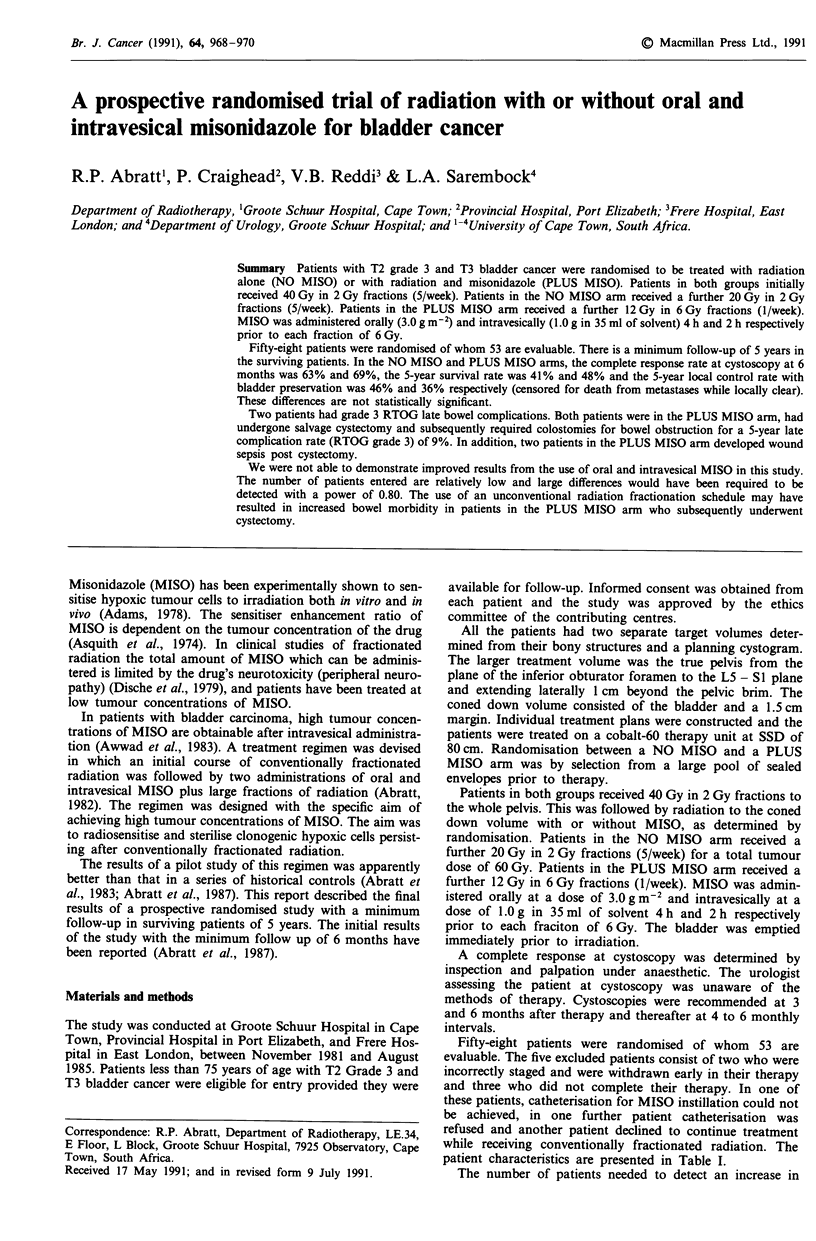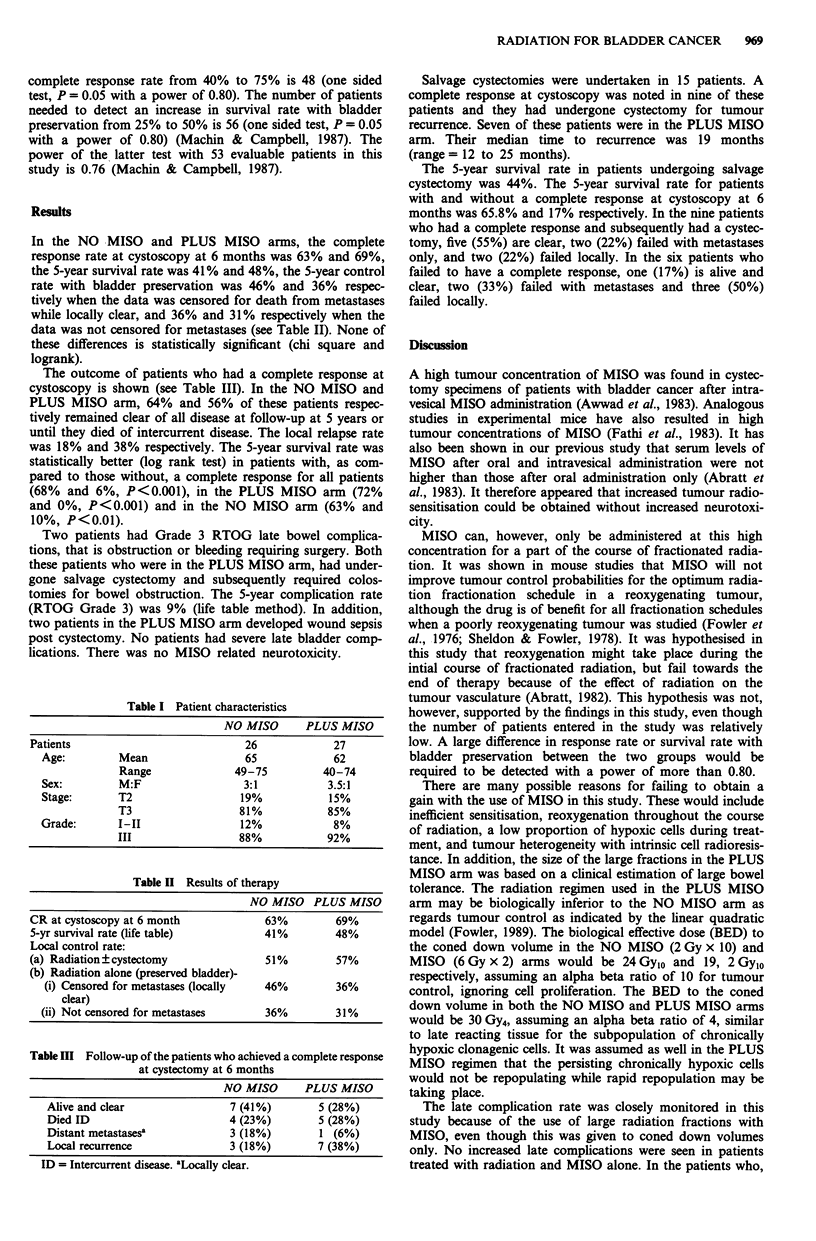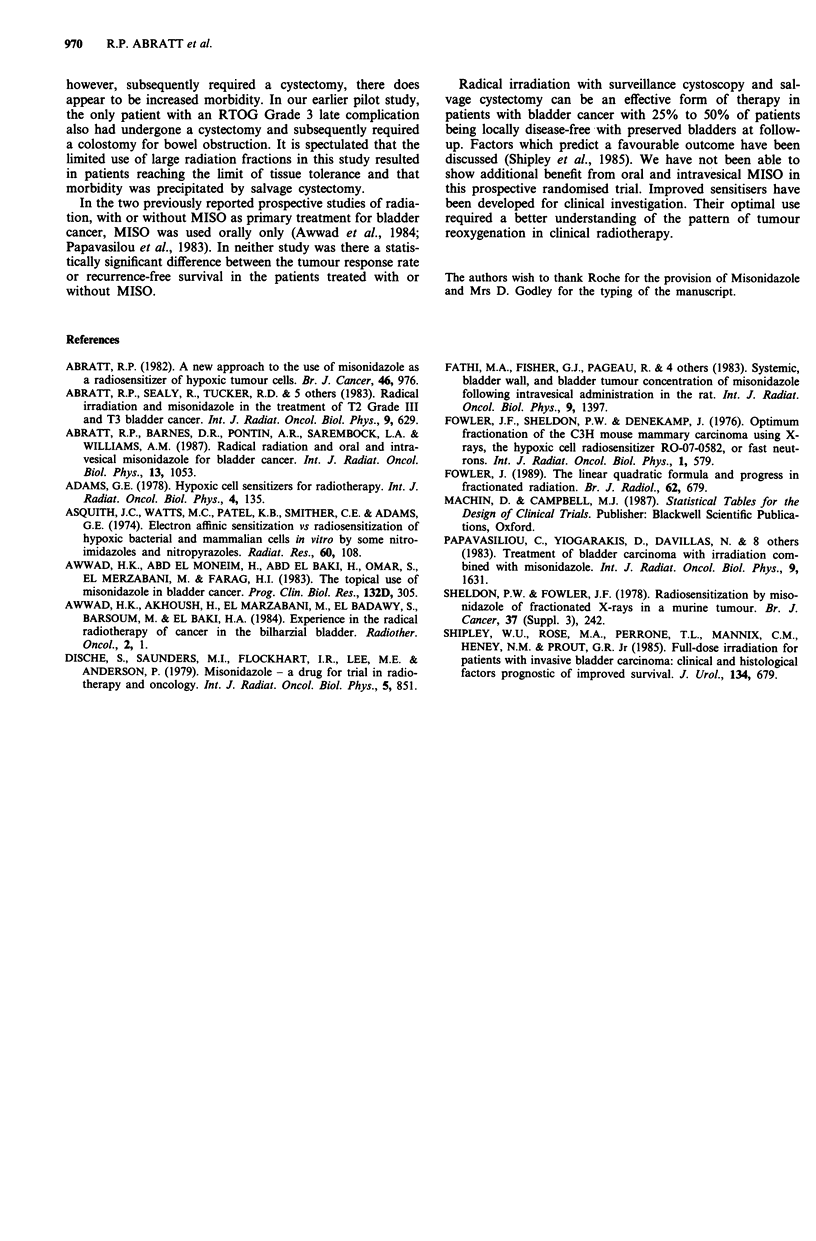# A prospective randomised trial of radiation with or without oral and intravesical misonidazole for bladder cancer.

**DOI:** 10.1038/bjc.1991.438

**Published:** 1991-11

**Authors:** R. P. Abratt, P. Craighead, V. B. Reddi, L. A. Sarembock

**Affiliations:** Department of Radiotherapy, Groote Schuur Hospital, Cape Town, South Africa.

## Abstract

Patients with T2 grade 3 and T3 bladder cancer were randomised to be treated with radiation alone (NO MISO) or with radiation and misonidazole (PLUS MISO). Patients in both groups initially received 40 Gy in 2 Gy fractions (5/week). Patients in the NO MISO arm received a further 20 Gy in 2 Gy fractions (5/week). Patients in the PLUS MISO arm received a further 12 Gy in 6 Gy fractions (1/week). MISO was administered orally (3.0 g m-2) and intravesically (1.0 g in 35 ml of solvent) 4 h and 2 h respectively prior to each fraction of 6 Gy. Fifty-eight patients were randomized of whom 53 are evaluable. There is a minimum follow-up of 5 years in the surviving patients. In the NO MISO and PLUS MISO arms, the complete response rate at cystoscopy at 6 months was 63% and 69%, the 5-year survival rate was 41% and 48% and the 5-year local control rate with bladder preservation was 46% and 36% respectively (censored for death from metastases while locally clear). These differences are not statistically significant. Two patients had grade 3 RTOG late bowel complications. Both patients were in the PLUS MISO arm, had undergone salvage cystectomy and subsequently required colostomies for bowel obstruction for a 5-year late complication rate (RTOG grade 3) of 9%. In addition, two patients in the PLUS MISO arm developed wound sepsis post cystectomy. We were not able to demonstrate improved results from the use of oral and intravesical MISO in this study. The number of patients entered are relatively low and large differences would have been required to be detected with a power of 0.80. The use of an unconventional radiation fractionation schedule may have resulted in increased bowel morbidity in patients in the PLUS MISO arm who subsequently underwent cystectomy.


Br. J. Cancer (1991), 64, 968–970 © Macmillan Press Ltd., 1991

### A prospective randomised trial of radiation with or without oral and intravesical misonidazole for bladder cancer

R.P. Abratt[1], P. Craighead[2], V.B. Reddi[3] & L.A. Sarembock[4]

*Department of Radiotherapy, [1]Groote Schuur Hospital, Cape Town; [2]Provincial Hospital, Port Elizabeth; [3]Frere Hospital, East London; and [4]Department of Urology, Groote Schuur Hospital; and [1–4]University of Cape Town, South Africa.*

**Summary** Patients with T2 grade 3 and T3 bladder cancer were randomised to be treated with radiation alone (NO MISO) or with radiation and misonidazole (PLUS MISO). Patients in both groups initially received 40 Gy in 2 Gy fractions (5/week). Patients in the NO MISO arm received a further 20 Gy in 2 Gy fractions (5/week). Patients in the PLUS MISO arm received a further 12 Gy in 6 Gy fractions (1/week). MISO was administered orally (3.0 g $m^{-2}$) and intravesically (1.0 g in 35 ml of solvent) 4 h and 2 h respectively prior to each fraction of 6 Gy.

Fifty-eight patients were randomised of whom 53 are evaluable. There is a minimum follow-up of 5 years in the surviving patients. In the NO MISO and PLUS MISO arms, the complete response rate at cystoscopy at 6 months was 63% and 69%, the 5-year survival rate was 41% and 48% and the 5-year local control rate with bladder preservation was 46% and 36% respectively (censored for death from metastases while locally clear). These differences are not statistically significant.

Two patients had grade 3 RTOG late bowel complications. Both patients were in the PLUS MISO arm, had undergone salvage cystectomy and subsequently required colostomies for bowel obstruction for a 5-year late complication rate (RTOG grade 3) of 9%. In addition, two patients in the PLUS MISO arm developed wound sepsis post cystectomy.

We were not able to demonstrate improved results from the use of oral and intravesical MISO in this study. The number of patients entered are relatively low and large differences would have been required to be detected with a power of 0.80. The use of an unconventional radiation fractionation schedule may have resulted in increased bowel morbidity in patients in the PLUS MISO arm who subsequently underwent cystectomy.

Misonidazole (MISO) has been experimentally shown to sensitise hypoxic tumour cells to irradiation both *in vitro* and *in vivo* (Adams, 1978). The sensitiser enhancement ratio of MISO is dependent on the tumour concentration of the drug (Asquith *et al.*, 1974). In clinical studies of fractionated radiation the total amount of MISO which can be administered is limited by the drug's neurotoxicity (peripheral neuropathy) (Dische *et al.*, 1979), and patients have been treated at low tumour concentrations of MISO.

In patients with bladder carcinoma, high tumour concentrations of MISO are obtainable after intravesical administration (Awwad *et al.*, 1983). A treatment regimen was devised in which an initial course of conventionally fractionated radiation was followed by two administrations of oral and intravesical MISO plus large fractions of radiation (Abratt, 1982). The regimen was designed with the specific aim of achieving high tumour concentrations of MISO. The aim was to radiosensitise and sterilise clonogenic hypoxic cells persisting after conventionally fractionated radiation.

The results of a pilot study of this regimen was apparently better than that in a series of historical controls (Abratt *et al.*, 1983; Abratt *et al.*, 1987). This report described the final results of a prospective randomised study with a minimum follow-up in surviving patients of 5 years. The initial results of the study with the minimum follow up of 6 months have been reported (Abratt *et al.*, 1987).

### Materials and methods

The study was conducted at Groote Schuur Hospital in Cape Town, Provincial Hospital in Port Elizabeth, and Frere Hospital in East London, between November 1981 and August 1985. Patients less than 75 years of age with T2 Grade 3 and T3 bladder cancer were eligible for entry provided they were available for follow-up. Informed consent was obtained from each patient and the study was approved by the ethics committee of the contributing centres.

All the patients had two separate target volumes determined from their bony structures and a planning cystogram. The larger treatment volume was the true pelvis from the plane of the inferior obturator foramen to the L5 – S1 plane and extending laterally 1 cm beyond the pelvic brim. The coned down volume consisted of the bladder and a 1.5 cm margin. Individual treatment plans were constructed and the patients were treated on a cobalt-60 therapy unit at SSD of 80 cm. Randomisation between a NO MISO and a PLUS MISO arm was by selection from a large pool of sealed envelopes prior to therapy.

Patients in both groups received 40 Gy in 2 Gy fractions to the whole pelvis. This was followed by radiation to the coned down volume with or without MISO, as determined by randomisation. Patients in the NO MISO arm received a further 20 Gy in 2 Gy fractions (5/week) for a total tumour dose of 60 Gy. Patients in the PLUS MISO arm received a further 12 Gy in 6 Gy fractions (1/week). MISO was administered orally at a dose of 3.0 g $m^{-2}$ and intravesically at a dose of 1.0 g in 35 ml of solvent 4 h and 2 h respectively prior to each fraciton of 6 Gy. The bladder was emptied immediately prior to irradiation.

A complete response at cystoscopy was determined by inspection and palpation under anaesthetic. The urologist assessing the patient at cystoscopy was unaware of the methods of therapy. Cystoscopies were recommended at 3 and 6 months after therapy and thereafter at 4 to 6 monthly intervals.

Fifty-eight patients were randomised of whom 53 are evaluable. The five excluded patients consist of two who were incorrectly staged and were withdrawn early in their therapy and three who did not complete their therapy. In one of these patients, catheterisation for MISO instillation could not be achieved, in one further patient catheterisation was refused and another patient declined to continue treatment while receiving conventionally fractionated radiation. The patient characteristics are presented in Table I.

The number of patients needed to detect an increase in

Correspondence: R.P. Abratt, Department of Radiotherapy, LE.34, E Floor, L Block, Groote Schuur Hospital, 7925 Observatory, Cape Town, South Africa.
Received 17 May 1991; and in revised form 9 July 1991.

complete response rate from 40% to 75% is 48 (one sided test, $P = 0.05$ with a power of 0.80). The number of patients needed to detect an increase in survival rate with bladder preservation from 25% to 50% is 56 (one sided test, $P = 0.05$ with a power of 0.80) (Machin & Campbell, 1987). The power of the latter test with 53 evaluable patients in this study is 0.76 (Machin & Campbell, 1987).

### Results

In the NO MISO and PLUS MISO arms, the complete response rate at cystoscopy at 6 months was 63% and 69%, the 5-year survival rate was 41% and 48%, the 5-year control rate with bladder preservation was 46% and 36% respectively when the data was censored for death from metastases while locally clear, and 36% and 31% respectively when the data was not censored for metastases (see Table II). None of these differences is statistically significant (chi square and logrank).

The outcome of patients who had a complete response at cystoscopy is shown (see Table III). In the NO MISO and PLUS MISO arm, 64% and 56% of these patients respectively remained clear of all disease at follow-up at 5 years or until they died of intercurrent disease. The local relapse rate was 18% and 38% respectively. The 5-year survival rate was statistically better (log rank test) in patients with, as compared to those without, a complete response for all patients (68% and 6%, $P<0.001$), in the PLUS MISO arm (72% and 0%, $P<0.001$) and in the NO MISO arm (63% and 10%, $P<0.01$).

Two patients had Grade 3 RTOG late bowel complications, that is obstruction or bleeding requiring surgery. Both these patients who were in the PLUS MISO arm, had undergone salvage cystectomy and subsequently required colostomies for bowel obstruction. The 5-year complication rate (RTOG Grade 3) was 9% (life table method). In addition, two patients in the PLUS MISO arm developed wound sepsis post cystectomy. No patients had severe late bladder complications. There was no MISO related neurotoxicity.

**Table I** Patient characteristics

| | | *NO MISO* | *PLUS MISO* |
|---|---|---|---|
| Patients | | 26 | 27 |
| Age: | Mean | 65 | 62 |
| | Range | 49–75 | 40–74 |
| Sex: | M:F | 3:1 | 3.5:1 |
| Stage: | T2 | 19% | 15% |
| | T3 | 81% | 85% |
| Grade: | I–II | 12% | 8% |
| | III | 88% | 92% |

**Table II** Results of therapy

| | *NO MISO* | *PLUS MISO* |
|---|---|---|
| CR at cystoscopy at 6 month | 63% | 69% |
| 5-yr survival rate (life table) | 41% | 48% |
| Local control rate: | | |
| (a) Radiation ± cystectomy | 51% | 57% |
| (b) Radiation alone (preserved bladder)- | | |
| (i) Censored for metastases (locally clear) | 46% | 36% |
| (ii) Not censored for metastases | 36% | 31% |

**Table III** Follow-up of the patients who achieved a complete response at cystectomy at 6 months

| | *NO MISO* | *PLUS MISO* |
|---|---|---|
| Alive and clear | 7 (41%) | 5 (28%) |
| Died ID | 4 (23%) | 5 (28%) |
| Distant metastases[a] | 3 (18%) | 1 (6%) |
| Local recurrence | 3 (18%) | 7 (38%) |

ID = Intercurrent disease. [a]Locally clear.

Salvage cystectomies were undertaken in 15 patients. A complete response at cystoscopy was noted in nine of these patients and they had undergone cystectomy for tumour recurrence. Seven of these patients were in the PLUS MISO arm. Their median time to recurrence was 19 months (range = 12 to 25 months).

The 5-year survival rate in patients undergoing salvage cystectomy was 44%. The 5-year survival rate for patients with and without a complete response at cystoscopy at 6 months was 65.8% and 17% respectively. In the nine patients who had a complete response and subsequently had a cystectomy, five (55%) are clear, two (22%) failed with metastases only, and two (22%) failed locally. In the six patients who failed to have a complete response, one (17%) is alive and clear, two (33%) failed with metastases and three (50%) failed locally.

### Discussion

A high tumour concentration of MISO was found in cystectomy specimens of patients with bladder cancer after intravesical MISO administration (Awwad *et al.*, 1983). Analogous studies in experimental mice have also resulted in high tumour concentrations of MISO (Fathi *et al.*, 1983). It has also been shown in our previous study that serum levels of MISO after oral and intravesical administration were not higher than those after oral administration only (Abratt *et al.*, 1983). It therefore appeared that increased tumour radiosensitisation could be obtained without increased neurotoxicity.

MISO can, however, only be administered at this high concentration for a part of the course of fractionated radiation. It was shown in mouse studies that MISO will not improve tumour control probabilities for the optimum radiation fractionation schedule in a reoxygenating tumour, although the drug is of benefit for all fractionation schedules when a poorly reoxygenating tumour was studied (Fowler *et al.*, 1976; Sheldon & Fowler, 1978). It was hypothesised in this study that reoxygenation might take place during the intial course of fractionated radiation, but fail towards the end of therapy because of the effect of radiation on the tumour vasculature (Abratt, 1982). This hypothesis was not, however, supported by the findings in this study, even though the number of patients entered in the study was relatively low. A large difference in response rate or survival rate with bladder preservation between the two groups would be required to be detected with a power of more than 0.80.

There are many possible reasons for failing to obtain a gain with the use of MISO in this study. These would include inefficient sensitisation, reoxygenation throughout the course of radiation, a low proportion of hypoxic cells during treatment, and tumour heterogeneity with intrinsic cell radioresistance. In addition, the size of the large fractions in the PLUS MISO arm was based on a clinical estimation of large bowel tolerance. The radiation regimen used in the PLUS MISO arm may be biologically inferior to the NO MISO arm as regards tumour control as indicated by the linear quadratic model (Fowler, 1989). The biological effective dose (BED) to the coned down volume in the NO MISO (2 Gy × 10) and MISO (6 Gy × 2) arms would be 24 $Gy_{10}$ and 19, 2 $Gy_{10}$ respectively, assuming an alpha beta ratio of 10 for tumour control, ignoring cell proliferation. The BED to the coned down volume in both the NO MISO and PLUS MISO arms would be 30 $Gy_4$, assuming an alpha beta ratio of 4, similar to late reacting tissue for the subpopulation of chronically hypoxic clonagenic cells. It was assumed as well in the PLUS MISO regimen that the persisting chronically hypoxic cells would not be repopulating while rapid repopulation may be taking place.

The late complication rate was closely monitored in this study because of the use of large radiation fractions with MISO, even though this was given to coned down volumes only. No increased late complications were seen in patients treated with radiation and MISO alone. In the patients who, however, subsequently required a cystectomy, there does appear to be increased morbidity. In our earlier pilot study, the only patient with an RTOG Grade 3 late complication also had undergone a cystectomy and subsequently required a colostomy for bowel obstruction. It is spectulated that the limited use of large radiation fractions in this study resulted in patients reaching the limit of tissue tolerance and that morbidity was precipitated by salvage cystectomy.

In the two previously reported prospective studies of radiation, with or without MISO as primary treatment for bladder cancer, MISO was used orally only (Awwad *et al.*, 1984; Papavasilou *et al.*, 1983). In neither study was there a statistically significant difference between the tumour response rate or recurrence-free survival in the patients treated with or without MISO.

Radical irradiation with surveillance cystoscopy and salvage cystectomy can be an effective form of therapy in patients with bladder cancer with 25% to 50% of patients being locally disease-free with preserved bladders at follow-up. Factors which predict a favourable outcome have been discussed (Shipley *et al.*, 1985). We have not been able to show additional benefit from oral and intravesical MISO in this prospective randomised trial. Improved sensitisers have been developed for clinical investigation. Their optimal use required a better understanding of the pattern of tumour reoxygenation in clinical radiotherapy.

The authors wish to thank Roche for the provision of Misonidazole and Mrs D. Godley for the typing of the manuscript.

### References

ABRATT, R.P. (1982). A new approach to the use of misonidazole as a radiosensitizer of hypoxic tumour cells. *Br. J. Cancer*, **46**, 976.

ABRATT, R.P., SEALY, R., TUCKER, R.D. & 5 others (1983). Radical irradiation and misonidazole in the treatment of T2 Grade III and T3 bladder cancer. *Int. J. Radiat. Oncol. Biol. Phys.*, **9**, 629.

ABRATT, R.P., BARNES, D.R., PONTIN, A.R., SAREMBOCK, L.A. & WILLIAMS, A.M. (1987). Radical radiation and oral and intravesical misonidazole for bladder cancer. *Int. J. Radiat. Oncol. Biol. Phys.*, **13**, 1053.

ADAMS, G.E. (1978). Hypoxic cell sensitizers for radiotherapy. *Int. J. Radiat. Oncol. Biol. Phys.*, **4**, 135.

ASQUITH, J.C., WATTS, M.C., PATEL, K.B., SMITHER, C.E. & ADAMS, G.E. (1974). Electron affinic sensitization *vs* radiosensitization of hypoxic bacterial and mammalian cells *in vitro* by some nitroimidazoles and nitropyrazoles. *Radiat. Res.*, **60**, 108.

AWWAD, H.K., ABD EL MONEIM, H., ABD EL BAKI, H., OMAR, S., EL MERZABANI, M. & FARAG, H.I. (1983). The topical use of misonidazole in bladder cancer. *Prog. Clin. Biol. Res.*, **132D**, 305.

AWWAD, H.K., AKHOUSH, H., EL MARZABANI, M., EL BADAWY, S., BARSOUM, M. & EL BAKI, H.A. (1984). Experience in the radical radiotherapy of cancer in the bilharzial bladder. *Radiother. Oncol.*, **2**, 1.

DISCHE, S., SAUNDERS, M.I., FLOCKHART, I.R., LEE, M.E. & ANDERSON, P. (1979). Misonidazole – a drug for trial in radiotherapy and oncology. *Int. J. Radiat. Oncol. Biol. Phys.*, **5**, 851.

FATHI, M.A., FISHER, G.J., PAGEAU, R. & 4 others (1983). Systemic, bladder wall, and bladder tumour concentration of misonidazole following intravesical administration in the rat. *Int. J. Radiat. Oncol. Biol. Phys.*, **9**, 1397.

FOWLER, J.F., SHELDON, P.W. & DENEKAMP, J. (1976). Optimum fractionation of the C3H mouse mammary carcinoma using X-rays, the hypoxic cell radiosensitizer RO-07-0582, or fast neutrons. *Int. J. Radiat. Oncol. Biol. Phys.*, **1**, 579.

FOWLER, J. (1989). The linear quadratic formula and progress in fractionated radiation. *Br. J. Radiol.*, **62**, 679.

MACHIN, D. & CAMPBELL, M.J. (1987). *Statistical Tables for the Design of Clinical Trials*. Publisher: Blackwell Scientific Publications, Oxford.

PAPAVASILIOU, C., YIOGARAKIS, D., DAVILLAS, N. & 8 others (1983). Treatment of bladder carcinoma with irradiation combined with misonidazole. *Int. J. Radiat. Oncol. Biol. Phys.*, **9**, 1631.

SHELDON, P.W. & FOWLER, J.F. (1978). Radiosensitization by misonidazole of fractionated X-rays in a murine tumour. *Br. J. Cancer*, **37** (Suppl. 3), 242.

SHIPLEY, W.U., ROSE, M.A., PERRONE, T.L., MANNIX, C.M., HENEY, N.M. & PROUT, G.R. Jr (1985). Full-dose irradiation for patients with invasive bladder carcinoma: clinical and histological factors prognostic of improved survival. *J. Urol.*, **134**, 679.